# Predicting Speech Intelligibility Based on Across-Frequency Contrast in Simulated Auditory-Nerve Fluctuations

**DOI:** 10.3813/aaa.919245

**Published:** 2018

**Authors:** Christoph Scheidiger, Laurel H. Carney, Torsten Dau, Johannes Zaar

**Affiliations:** 1) Hearing Systems group, Department of Electrical Engineering, Technical University of Denmark, 2800 Kgs.Lyngby.; 2) Departments of Biomedical Engineering, and Neurobiology and Anatomy, University of Rochester, Rochester, New York 14642

## Abstract

The present study proposes a modeling approach for predicting speech intelligibility for normal-hearing (NH) and hearing-impaired (HI) listeners in conditions of stationary and fluctuating interferers. The model combines a non-linear model of the auditory periphery with a decision process that is based on the contrast across characteristic frequency (CF) after modulation analysis in the range of the fundamental frequency of speech. Specifically the short-term across-CF correlation between noisy speech and noise alone is assumed to be inversely related to speech intelligibility. The model provided highly accurate predictions for NH listeners as well as largely plausible effects in response to changes in presentation level. Furthermore, the model could account for some of the main features in the HI data solely by adapting the peripheral model using a simplistic interpretation of the listeners’ hearing thresholds. The model’s predictive power may be substantially improved by refining the interpretation of the HI listeners’ profiles and the model may thus p rovide a valuable basis for quantitatively modeling effects of outer hair-cell and inner hair-cell loss on speech intelligibility.

## 1. Introduction

Numerous speech intelligibility (SI) models have been proposed to predict the ability of normal-hearing (NH) listeners to understand speech in adverse listening conditions. These models typically employ an auditory-inspired spectral decomposition and assume SI to be related to the signal-to-noise ratio (SNR; e.g. SII [1]) or to the correlation between the clean and the noisy speech (e.g. STOI [2]). Some of the most powerful and versatile SI models additionally consider a modulation-frequency selective analysis and relate SI to the SNR in the envelope domain (SNR_env_; e.g. mr-sEPSM [3]) or to the correlation between the clean and the noisy speech in the modulation domain (e.g. sEPSM^corr^ [4]). Despite their strong predictive power for data from NH listeners, most current SI models are functionally limited in their ability to predict the detrimental effects of hearing impairment on SI due to their strongly simplified linear representation of the highly non-linear auditory periphery.

Non-linear models of the auditory periphery may be used in place of the simplified linear models. One such model is an auditory-nerve (AN) model that has been developed to describe the temporal properties of auditory-nerve spike trains in cats and other species [5, 6, 7]. It can functionally account for level effects as well as for outer hair-cell (OHC) and inner hair-cell (IHC) loss. Some attempts have been made to predict SI using the AN model as a front end and comparing the internal representations of the stimulus to a clean-speech template [8, 9]. The models showed promising predictions of consonant-vowel and word recognition, but not sentence recognition.

**Figure 1.**
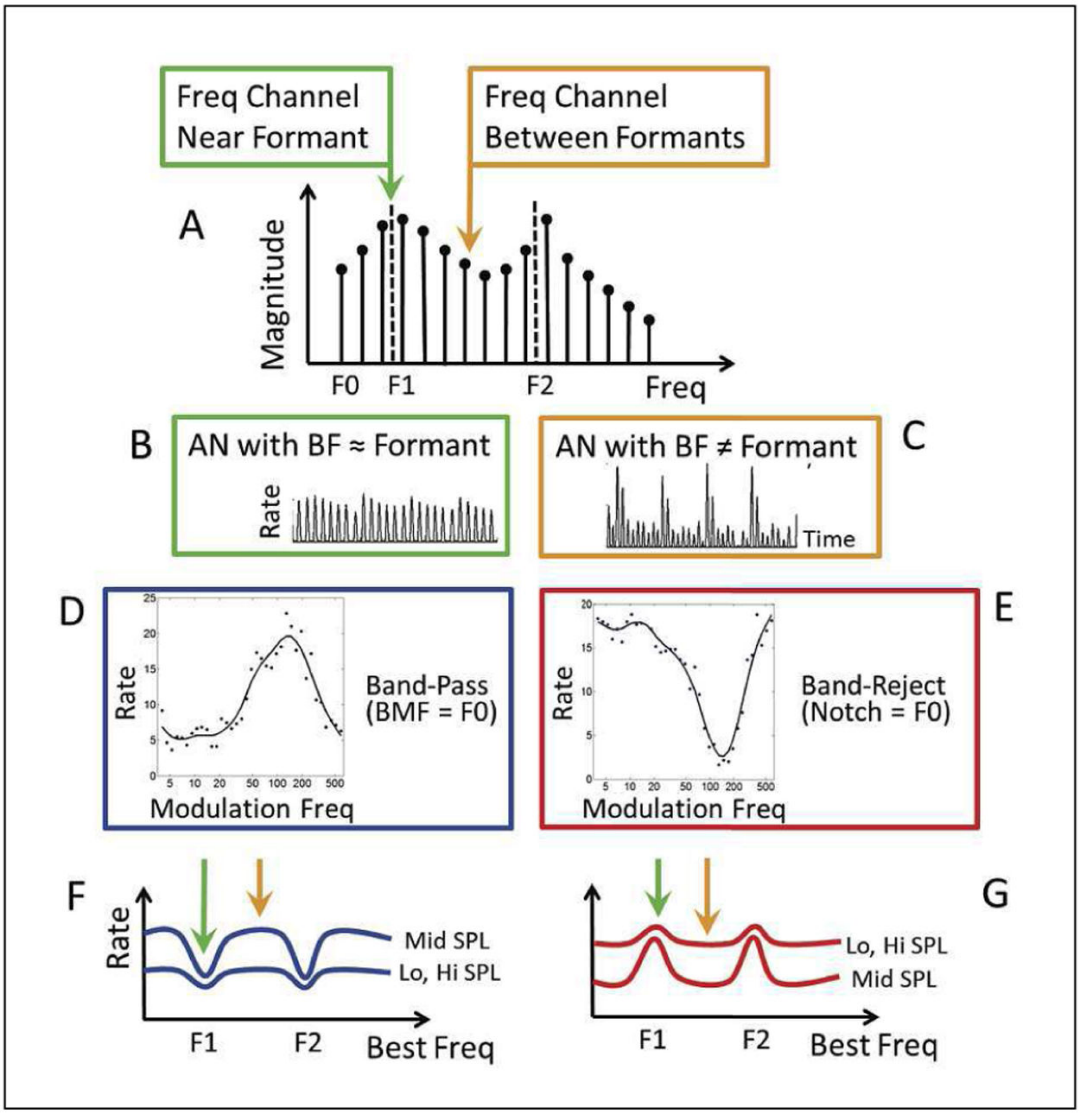
Underlying hypothesis of vowel coding in the midbrain. Reprinted from [10].

In a recent study [10], Carney *et al*. proposed a model of vowel coding in the midbrain, which was shown to be robust over a wide range of sound levels as well as in background noise. The model is based on the non-linear behaviour of the IHCs in the AN model, which are saturated at characteristic frequencies (CFs) close to the high-level vowel formants, resulting in an almost uniform AN response amplitude over time (Figure 1, panel B), whereas they respond more linearly to the low-level spectral components at CFs between formants, yielding a response pattern with strong amplitude fluctuations around the fundamental frequency (F_0_) due to the interaction (“beating”) of the vowel harmonics (Figure 1, panel C). Based on the behaviour of inferior-colliculus (IC) neurons, many of which exhibit band-pass tuning to amplitude modulations [10], the contrast in the amplitude of these rate fluctuations is enhanced using a band-pass filter with a best modulation frequency (BMF) in the F_0_ range (Figure 1, panel D). As a result, the simulated IC rates show high activity for CFs in between vowel formants and steep notches for CFs close to vowel formants (Figure 1, panel F). This pattern is hypothesized to provide a salient across-CF contrast cue for detecting and discriminating voiced speech sounds.

The present study adopted the approach from [10] to predict intelligibility for NH and hearing-impaired (HI) listeners for sentences in different types of interferers. In contrast to earlier AN-model-based SI models [8, 9], the noise alone was chosen as a reference signal instead of the clean speech, inspired by the decision metric of the mr-sEPSM model (SNR_env_, [3]). A short-term correlation metric was applied to quantify the similarity of the across-CF contrast patterns of noisy speech and noise alone.

## 2. Model description

**Figure 2.**
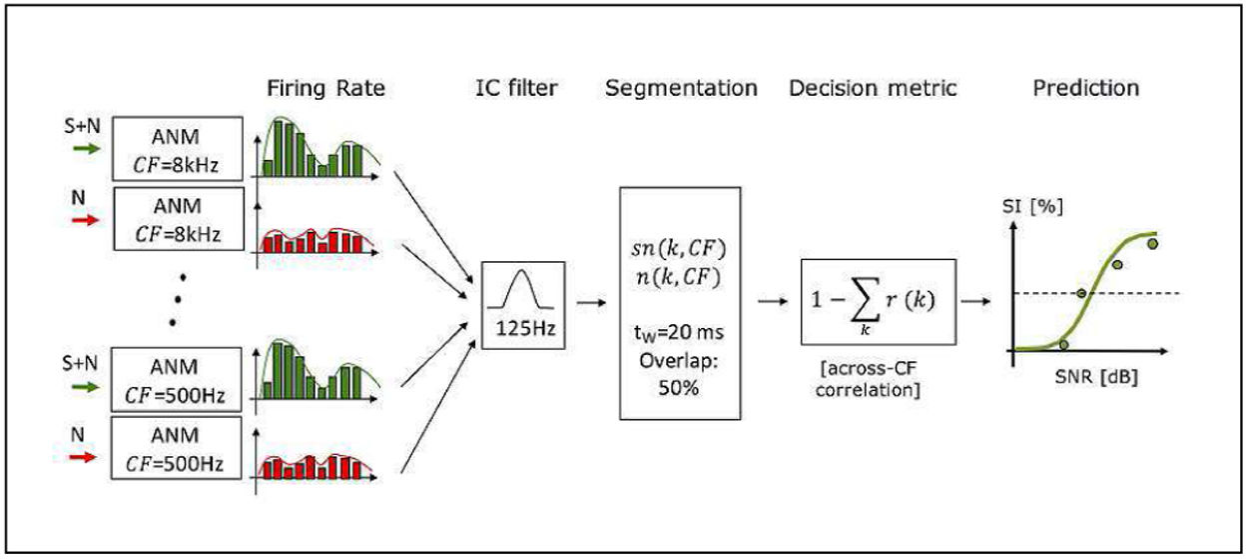
Structure of the proposed model.

The proposed model takes the noisy speech signal (SN) and the noise alone (N) as inputs. It consists of three main stages (see Figure 2): (i) a peripheral front end, represented by the AN model [7], (ii) a simplistic midbrain model that enhances the firing patterns according to [10] (see Figure 1), and (iii) a decision stage, which performs a short-term correlation analysis and relates the outcome to SI.

The front end of the model consists of the AN model [7], which describes the auditory periphery in terms of peripheral frequency tuning and several non-linear aspects of cochlear and hair cell responses. The model allows adjustment of the functioning of the OHC and IHC to simulate hearing losses. Here, the instantaneous firing rate at the output of the IHC-AN synapse model was considered for further processing, omitting the final spike-time generation stage of the AN model. 13 CFs logarithmically spaced between 0.5 and 8 kHz were considered. For each CF, 50 fibers were simulated using 60% high, 20% medium, and 20% low spontaneous-rate fibers (HSR, MSR, and LSR, respectively; proportions taken from [8]) and the resulting instantaneous firing rates were averaged across fibers.

In the midbrain stage of the model, the modulation transfer functions of the IC neurons were represented as a single band-pass filter with a Q of 1, centred at *f_c_* = 125 Hz, i.e., close to the F_0_ of the target speaker (see below). The average instantaneous firing rate obtained for each CF was filtered and the output of the filter was segmented into 20-ms time frames in 10-ms steps (i.e., using 50% overlap). Within each time frame, the values were squared and averaged across temporal samples. The noisy speech and the noise alone were thus represented as functions of time segment *k* and CF as *sn*(*k, CF*) and *n*(*k, CF*), respectively.

In the decision stage of the model, the across-CF correlation coefficent *r*(*k*) between *sn*(*k, CF*) and *n*(*k, CF*) was computed for each segment *k* and eventually averaged across segments, yielding $\bar{r}$. As $\bar{r}$ reflects the qualitative *similarity* between noisy speech and noise alone, it was assumed to be inversely related to SI. To obtain a decision metric that was directly related to SI, the final decision metric was calculated as $d=1-\bar{r}$, reflecting a qualitative *dissimilarity* metric. Finally, *d* was converted to SI according to *SI* (*SNR*) = $\frac{100\%}{1+e^{a_{1}d(SNR)+a_{2}}}$. The parameters *a*_1_ and *a*_2_ were estimated once based on a fitting condition with known SI scores using non-linear regression.

## 3. Method

Data collected from five NH listeners (between 24 and 33 years) were taken from [3]. Data collected from 13 HI listeners (between 51 and 72 years) were taken from [11]. Both studies used natural meaningful Danish five-word sentences, spoken by a male speaker with an average F_0_ of 119 Hz, and measured speech reception thresholds (SRTs) using an adaptive procedure. The speech level was set to 65 dB sound pressure level (SPL) for the NH listeners and to 80 dB SPL for the HI listeners. Three interferers were used: (i) Speech-shaped noise (SSN), a stationary masker with a long-term spectrum identical to the average spectrum of all sentences, (ii) an 8-Hz sinusoidally amplitude-modulated (SAM) SSN, and (iii) the international speech test signal (ISTS), a largely unintelligible signal that consists of randomly concatenated syllables taken from multiple recordings spoken by different female speakers in various languages (average F_0_: 207 Hz).

Ten sentences of the speech corpus were chosen for the model simulations. All simulation results were averaged across these ten sentences in order to obtain stable predictions. The simulated input SNRs ranged from −21 dB to 12 dB in 3-dB steps, covering the SRTs obtained for all listeners and conditions. The level of the noise alone reference signal was adjusted to have the same level as the noisy speech.

In the *NH operation mode*, the standard NH configuration of the AN model was used to predict the SRTs measured for NH listeners. Model predictions were obtained for speech levels of 50, 65, and 80 dB SPL, with 65 dB SPL corresponding to the speech level used in the experiment. The remaining levels were used to investigate the size and trends of level effects in the model predictions. The decision metric was converted to SI as described in Sec. 2 with parameters fitted based on the SSN condition and a speech level of 65 dB SPL using the NH AN-model configuration. The SRTs were obtained as the 50-% points on the predicted psychometric functions.

In the *HI operation mode*, the audiograms of the two ears of the 13 HI listeners (Table 1, p. 1657; [11]) were used to determine the OHC and IHC loss factors in the AN model. It was assumed that two thirds of the total hearing loss were related to OHC loss and one third to IHC loss [8, 9]. Model predictions were obtained for both ears of each listener and the better ear in terms of the predicted SI was chosen for each SNR. The model predictions were obtained at a speech level of 80 dB SPL, the same level at which the SRT data were measured [11]. The decision metric was converted to SI scores and SRTs using the same procedure and fitting parameters as before.

## 4. Results

**Figure 3.**
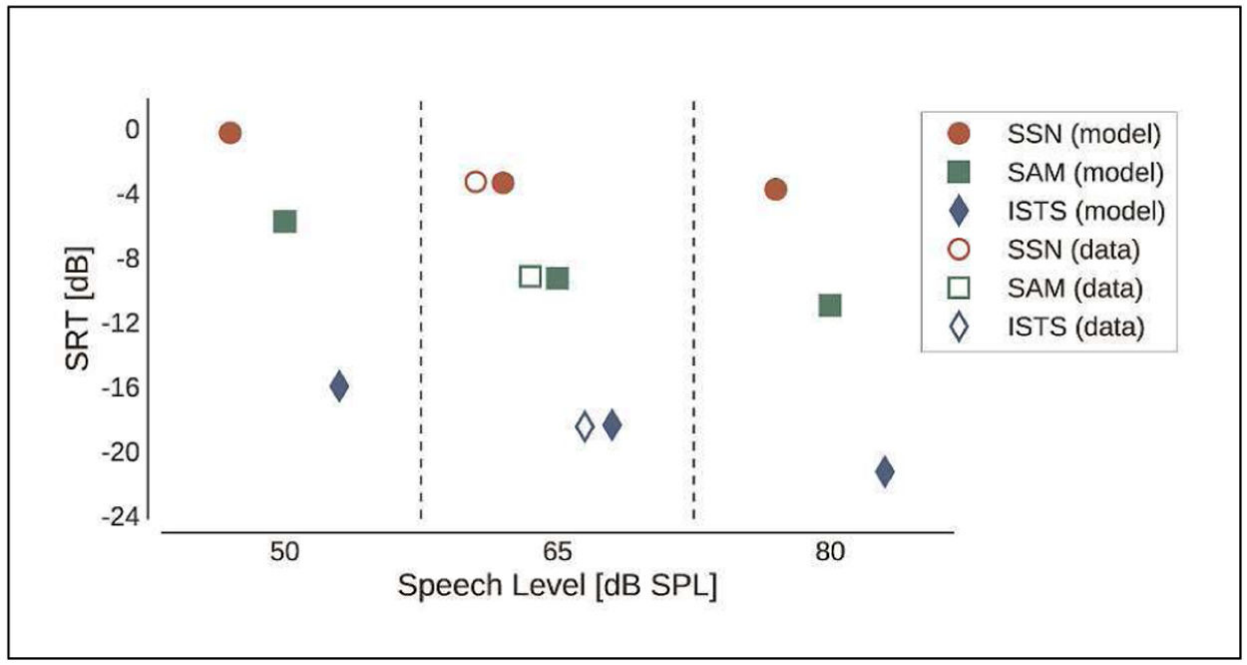
SRTs measured for NH listeners (open symbols) at a speech level of 65 dB SPL and SRTs predicted by the model (filled symbols) assuming speech levels of 50, 65, and 80 dB SPL (left to right).

Figure 3 shows the SRTs measured for the NH listeners (open symbols, [3]), which decrease (i.e., SI increases) from SSN (red circles) to SAM (green squares) to ISTS (blue diamonds). The model predictions using the NH operation mode (filled symbols) are shown for each speech level. The model accounted accurately for the data obtained at 65 dB SPL. For the speech level of 50 dB SPL, where no reference data were available, the model predicted slightly higher SRTs than for 65 dB SPL, reflecting lower SI. While this might be considered a plausible effect of limited audibility, this is not necessarily expected at the above-threshold speech level of 50 dB SPL. For the speech level of 80 dB SPL, the predicted SRTs were similar to the ones predicted for 65 dB SPL for SSN but lower (indicating higher SI) for the fluctuating interferers (SAM and ISTS). This is in good agreement with the corresponding literature.

**Figure 4.**
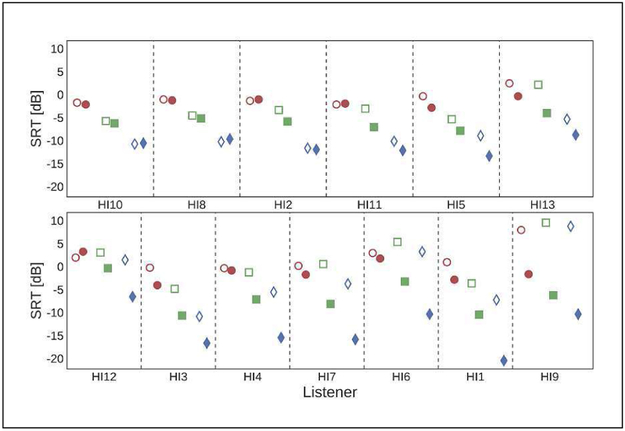
Measured and predicted SRTs for each HI listener, ordered from top left to bottom right according to decreasing model prediction accuracy. The symbols are as for Figure 3.

Figure 4 depicts the SRTs measured for the individual HI listeners (open symbols, [11]) and the corresponding model predictions (filled symbols), sorted according to model prediction accuracy. The model could account to a large extent for the general upward shift in SRTs and for the reduced spread in SRTs across noise conditions, capturing the main effects for about half of the HI listeners. However, for some listeners (e.g., HI9, HI1, HI6, HI7, HI4), the model predicted much lower SRTs than observed, in particular for the ISTS and, to some extent, the SAM condition. A condition-specific analysis revealed that the model performance was best for the SSN condition, followed by SAM and ISTS (mean average errors of 2.19, 5.48, and 7.12 dB, respectively). The condition-specific across-listener correlations between measured and predicted SRTs were moderately positive (0.3 ≤ *r* ≤ 0.5) but not significant (*p* > 0.05). However, after exclusion of the outlier test subject HI9 the correlations for SSN and SAM were significant (*r* ≥ 0.6, *p* < 0.05) while the correlation for ISTS remained low (*r* = 0.3, *p* > 0.05). The overall correlation between all measured and predicted SRTs was highly significant (*r* = 0.58, *p* < 0.001).

## 5. Discussion

The proposed model accounted accurately for NH listeners’ SRTs and also partly predicted plausible effects of presentation level, which is consistent with the results reported in [10]. Furthermore, the model accurately predicted SRTs for about half of the HI listeners. However, it also overestimated speech intelligibility in the conditions with fluctuating interferers (SAM and ISTS) for HI listeners who exhibited very little or no masking release.

Crucially, the model was fitted only once based on data for NH listeners and speech in SSN at a level of 65 dB SPL (as used in the corresponding experiment). All predictions were then obtained with this “frozen” transformation function, irrespective of the interferer, speech level, or hearing loss. Achieving accurate predictions for individual HI listeners in various conditions based on such a generic fitting constitutes a challenging test for a SI model, as the only effects of hearing loss the model was “allowed” to take into account were the OHC and IHC loss parameters in the AN-model front end.

The error and correlation analyses revealed that, while the overall trends across HI listeners and conditions were well-represented in the model, the variability across HI listeners within each condition was captured only to some extent, indicating a lack of individualization. Additionally, the better-ear pure-tone average showed higher condition-specific correlations with the SRTs (0.64 ≤ *r* ≤ 0.87, *p* < 0.05) than the predicted SRTs. Considering the simplistic representation of the hearing loss in the model (assuming 2/3 OHC loss and 1/3 IHC loss) this is not unexpected. Further investigations are required to determine the influence of different OHC versus IHC loss ratios and their influence on the SI predictions. The underlying principle of stable rate patterns considered here is heavily based on IHC transduction (cf. [7, 12]); it is therefore expected that the predicted SI worsens with increasing IHC loss. The model might thus also be interesting for investigating the effects of damaged IHCs and synapses (see also [12] for review).

In contrast to most SI models for NH [1, 2, 3, 4], which apply simplified linear simulations of the auditory periphery followed by an independent channel-by-channel signal analysis, the proposed model combines a non-linear peripheral model with a decision process based on across-frequency fluctuation patterns. In line with [10, 12], this approach yields promising predictions of SI for HI listeners, suggesting that these patterns might actually play a role in speech perception. Given its use of fluctuation cues in the F_0_ range, model performance will vary for different types of speech (e.g. whispered or noise-vocoded speech). The weaker fluctuation cues elicited by these stimuli are consistent with lower intelligibility and greater susceptibility to maskers; future studies are required to quantify these effects. Lastly, the use of a “noise alone” reference signal limits the model to conditions where the noise signal is well defined. It may thus seem desirable to use a clean-speech reference instead; however, earlier attempts with a clean-speech reference were discouraging. Consistent with the SNR_env_ concept (e.g. [3]), the dissimilarity between noisy speech and noise alone was thus found to be superior in the considered framework.

## References

[1] ANSI: ANSI S3.5-1997. Methods for calculation of the speech intelligibility index. American National Standards Institute, New York, 1997.

[2] Taal CH, Hendriks RC, Heusdens R, Jensen J: An algorithm for intelligibility prediction of time-frequency weighted noisy speech. IEEE Trans. Audio Speech Lang. Process 19(7) (2011) 2125–2136.

[3] Jørgensen S, Ewert SD, Dau T: A multi-resolution envelope-power based model for speech intelligibility. J Acoust Soc Am 134(1) (2013) 436–446. 23862819 10.1121/1.4807563

[4] Relaño-Iborra H, May T, Zaar J, Scheidiger C, Dau T: Predicting speech intelligibility based on a correlation metric in the envelope power spectrum domain. J Acoust Soc Am 140(4) (2016) 2670–2679. 27794330 10.1121/1.4964505

[5] Carney LH: A model for the responses of low-frequency auditory-nerve fibers in cat. J Acoust Soc Am 93(1) (1993) 401–417. 8423257 10.1121/1.405620

[6] Zilany MSA, Bruce IC, Nelson PC, Carney LH: A phenomenological model of the synapse between the inner hair cell and auditory nerve: long-term adaptation with power-law dynamics. J Acoust Soc Am 126(5) (2009) 2390–2412. 19894822 10.1121/1.3238250 PMC2787068

[7] Zilany MSA, Bruce IC, Carney LH: Updated parameters and expanded simulation options for a model of the auditory periphery. J Acoust Soc Am 135(1) (2014) 283–286. 24437768 10.1121/1.4837815 PMC3985897

[8] Zilany MSA, Bruce IC: Predictions of Speech Intelligibility with a Model of the Normal and Impaired Auditory-periphery. 3rd Int. IEEE/EMBS Conf. on Neural Engineering IEEE (2007) 481–485.

[9] Bruce IC, Leger AC, Moore BCJ, Lorenzi C: Physiological prediction of masking release for normal-hearing and hearing-impaired listeners. Proc. of Meetings on Acoustics 19(1) (2013) 050178.

[10] Carney LH, Li T, McDonough JM: Speech coding in the brain: representation of vowel formants by midbrain neurons tuned to sound fluctuations. eNeuro 2(4) (2015) 1–12. 10.1523/ENEURO.0004-15.2015 PMC4596011 26464993

[11] Christiansen C, Dau T: Relationship between masking release in fluctuating maskers and speech reception thresholds in stationary noise. J Acoust Soc Am 132(3) (2012) 1655–1666. 22978894 10.1121/1.4742732

[12] Carney LH: Supra-threshold hearing and fluctuation profiles: implications for sensorineural and hidden hearing loss. J Assoc Res Otolaryngol. (2018) 1–22. 29744729 10.1007/s10162-018-0669-5 PMC6081887

